# Long-Term Outcomes of Coronary Artery Bypass Grafting With the Combination of Free Right Internal Mammary Artery and Modified Proximal Anastomosis: A Retrospective Study

**DOI:** 10.1093/icvts/ivag169

**Published:** 2026-06-16

**Authors:** Shota Yasuda, Tomoki Cho, Shotaro Kaneko, Makoto Ikematsu, Tomoyuki Minami, Keiji Uchida, Aya Saito

**Affiliations:** Cardiovascular Center, Yokohama City University Medical Center, Yokohama, Kanagawa 232-0024, Japan; Cardiovascular Center, Yokohama City University Medical Center, Yokohama, Kanagawa 232-0024, Japan; Cardiovascular Center, Yokohama City University Medical Center, Yokohama, Kanagawa 232-0024, Japan; Cardiovascular Center, Yokohama City University Medical Center, Yokohama, Kanagawa 232-0024, Japan; Department of Surgery, Yokohama City University, Yokohama, Kanagawa 236-0004, Japan; Cardiovascular Center, Yokohama City University Medical Center, Yokohama, Kanagawa 232-0024, Japan; Department of Surgery, Yokohama City University, Yokohama, Kanagawa 236-0004, Japan

**Keywords:** right internal mammary artery, free graft, proximal anastomosis, Pouch technique

## Abstract

**Objectives:**

When a right internal mammary artery (IMA) is employed as a free graft for aorta-coronary bypass, the small diameter of it may pose technical challenges at the proximal anastomosis. To address this, we developed a dome-shaped configuration of the proximal right IMA—termed “Pouch technique”—prior to anastomosis to the ascending aorta, aiming to reduce the risk of anastomotic stenosis. This study evaluates the long-term outcomes of coronary artery bypass grafting (CABG) with the combination of free right IMA graft and this technique.

**Methods:**

Of 834 patients who underwent CABG at our institution between 2014 and 2024, 161 received a free right IMA graft. Among these, 145 patients were included in the analysis. All included patients underwent proximal anastomosis using Pouch technique. Graft patency was primarily assessed by coronary computed tomography angiography at 1, 5, and 10 years postoperatively.

**Results:**

During the follow-up period, 6 deaths were recorded including the 2 early postoperative deaths. Major adverse cardiac and cerebrovascular events occurred in 11 patients. Graft-related events were observed in 7 patients. At 10-year follow-up, overall survival and freedom from major adverse cardiac and cerebrovascular events were 93% and 82%, respectively. Cumulative incidence of graft events is 12%.

**Conclusions:**

The combination of free right IMA graft and Pouch technique demonstrated favourable long-term outcomes with a low incidence of proximal anastomotic complications.

## Graphical abstract

**Figure:**
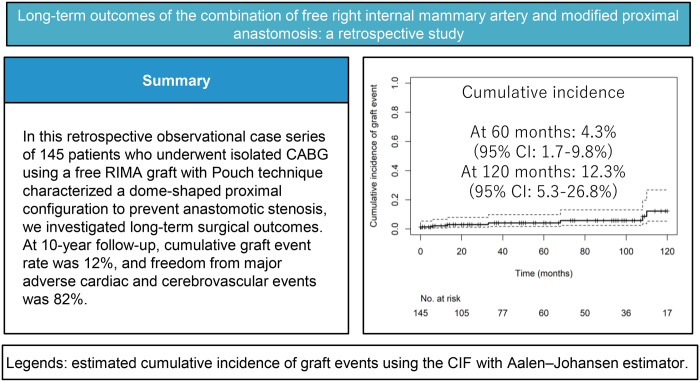

## INTRODUCTION

The right internal mammary artery (RIMA) is commonly employed as a secondary arterial conduit in coronary artery bypass grafting (CABG) following the left internal mammary artery (LIMA), particularly in younger patients. The clinical benefits of RIMA grafting have been well documented,^1^^,^^2^ recommended as a Class IIa indication in the 2018 ESC/EACTS guidelines as well,^3^ although its use has been associated with a relatively higher incidence of sternal wound infections.^4^

When used as an *in situ* graft, RIMA presents anatomical limitations that may hinder its ability to reach target coronary vessels. For the right coronary artery (RCA), it often fails to reach the posterior descending branch. For the left coronary system, while it may reach the proximal segment of left anterior descending artery (LAD), it typically cannot extend to the distal portion. Additionally, when the RIMA courses posterior to the sternum, it may increase the risk of injury during potential future resternotomy. Although there have been reports of successful revascularization of the circumflex artery using an *in situ* RIMA,^5^^,^^6^ the graft must pass the transverse sinus, which increases technical complexity.^7^ Moreover, it may not reach posterolateral branches due to anatomical limitations similar to posterior descending branch. Even when the graft reaches the intended coronary target, anastomosis at the distal end where the vessel is thinner may compromise anastomotic integrity.

Utilizing the RIMA as a free graft can overcome these anatomical constraints, and its efficacy has been demonstrated in several studies.^8–10^ However, compared to saphenous vein graft (SVG), the RIMA has a smaller diameter, which introduces technical difficulties at the proximal anastomosis site. Specifically, achieving an optimal anastomotic shape and height is challenging when the RIMA is anastomosed to an aortic opening sized for SVG. Downsizing the aortic opening to match the RIMA diameter may increase technical complexity and raise concerns regarding adequate graft flow.

To address these limitations, we developed a novel proximal anastomotic technique that preserves a dome-shaped configuration of the RIMA prior to its attachment to the ascending aorta—referred to as the “Pouch technique.”^11^ This study aims to evaluate the long-term outcomes of CABG with the combination of free RIMA and this technique.

## METHODS

### Ethical considerations

This study was conducted in accordance with the Declaration of Helsinki and the ethical guidelines for clinical research involving human subjects. Approval was obtained from the Ethics Committee of Yokohama City University (Approval No. F241100010, December 19, 2024). Data collected from research participants are protected in accordance with the principles outlined in the World Medical Association’s Taipei Declaration. The Ethics Committee has reviewed and approved the database protocol and provides ongoing oversight to ensure compliance. Given the retrospective nature of this study, the requirement for individual informed consent was waived. Instead, an explanatory statement was posted on the institutional website, and patients were given the opportunity to opt out of participation. It should be noted that the following ethical concerns were identified: at the time of induction of this technique, it was regarded as a minor modification within the scope of standard surgical practice and thus was not subject to formal ethical review or individual informed consent under our institutional policies.

### Patient cohort

Between 2014 and 2024, a total of 834 patients underwent CABG at our institution. Free RIMA grafting was performed in 161 cases, of which 145 patients who received proximal anastomosis to the ascending aorta using the Pouch technique were included in this study. Five cases with concomitant procedures such as valve surgery were excluded in the analysis.

### Selection criteria of patients and graft configuration

Indications for RIMA grafting included patients under 75 years of age and scheduled elective surgery. Patients with poorly controlled diabetes, extreme obesity, severe chronic obstructive pulmonary disease, prior mediastinal radiation therapy, or long-term high-dose steroid use were excluded according to the reported selection criteria.^12^ Patients with advanced peripheral arterial disease with RIMA-dependent collateral flow, and prior sternotomy were also excluded. However, in younger patients with poorly controlled diabetes, RIMA was often used following intensive preoperative management, including educational hospitalization.

Free RIMA grafting was planned in patients with a structurally healthy ascending aorta confirmed by preoperative computed tomography (CT). However, in selected cases, the preoperative plan was modified, and the free RIMA was used as a composite graft to reduce the time required for graft modification. In all other cases, a free RIMA was anastomosed to the ascending aorta using Pouch technique. We did not perform an intention-to-treat analysis for this study.

### Measurement of proximal RIMA diameter

Preoperative CT scans were used to measure the RIMA diameter at the level of the innominate vein. Measurements were independently performed by 2 surgeons, and gender-based differences in diameter were analysed.

### Surgical procedure

The RIMA was harvested using electrocautery in skeletonized fashion. Branches were divided using clips or bipolar coagulation forceps. The proximal segment was transected at the level of the right subclavian vein using triple clips, and the distal segment using double clips. Following harvest, the graft was immersed in a solution of verapamil and nitroglycerine (pH 7.4) to prevent intraoperative spasm.^13^

**Figure 1.**
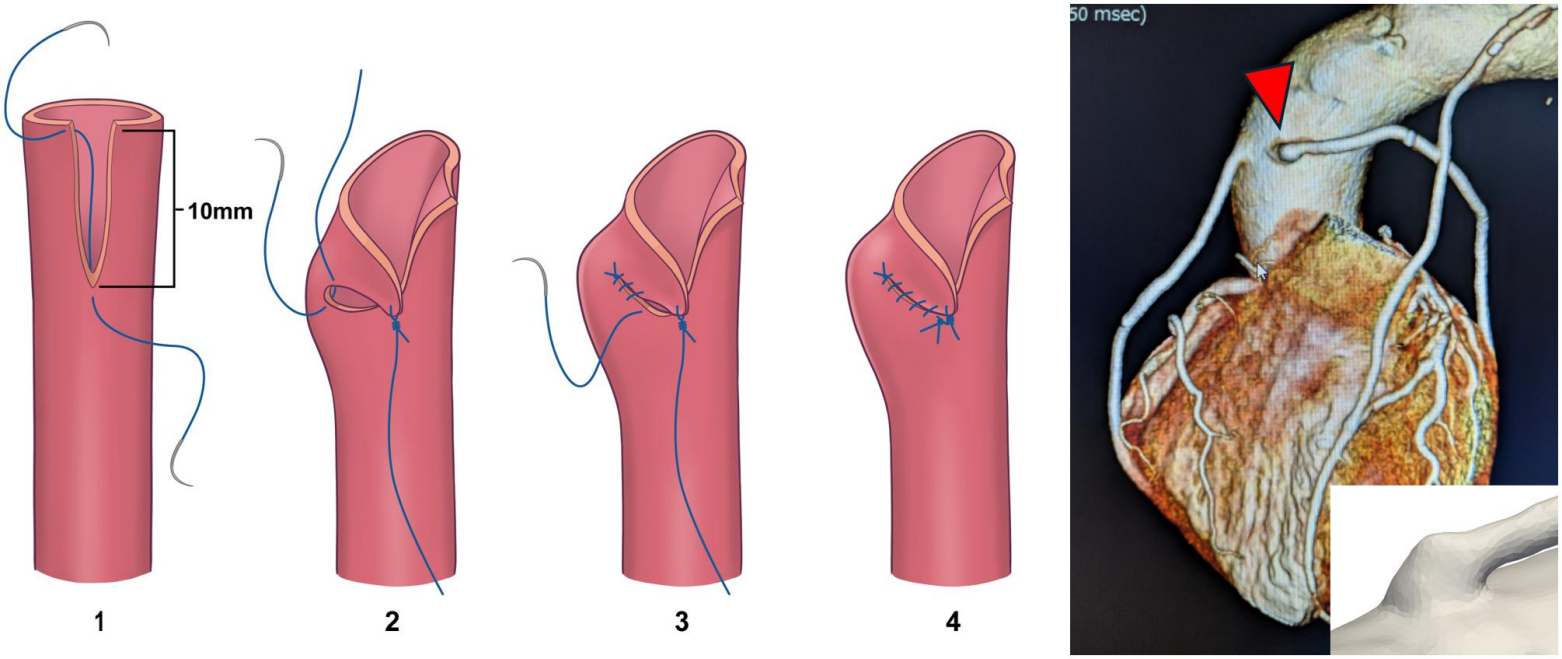
Graft Configuration Procedure and Images of Pouch Technique. (Left) The proximal stump of the internal thoracic artery was incised longitudinally for 10 mm. One edge of the incision was sutured to the opposite end using 7-0 polypropylene (1 and 2), and the folded sideline was closed with a continuous running suture (3 and 4). (Right) 3-Dementional CT scan image of free RIMA graft with magnified image of the proximal anastomosis using Pouch technique

The Pouch technique was performed prior to proximal anastomosis.^11^ Briefly, the proximal stump of the internal mammary artery (IMA) was incised longitudinally for 10 mm. One edge of the incision was sutured to the opposite end using 7-0 polypropylene, and the folded sideline was closed with a continuous running suture (**Figure 1**, **Video 1**).

Systemic heparinization was administered prior to transection of RIMA, which was performed either on-pump with cardioplegic arrest or off-pump. Distal anastomoses were completed first using an end-to-side technique with 8-0 polypropylene. The aortic anastomotic site was created using a 4 mm punch (CleanCut, Quest Medical, Inc.). For off-pump procedures, a 3.8 mm Heartstring III device (Getinge AB) was used. Anastomosis to the aorta was performed with 6-0 polypropylene sutures. Fibrin glue was applied to the anastomotic site to minimize bleeding. Prior to chest closure, graft flow was assessed using a transit-time flow measurement device (Veri Q, Medistim ASA), evaluating mean graft flow, pulsatility index (PI), and diastolic flow fraction.

### Postoperative follow-up

Annual follow-up evaluations were conducted. Graft patency was assessed via coronary CT angiography at 1, 5, and 10 years postoperatively as a part of routine follow-up care. In patients with contraindications to contrast media (eg, chronic kidney disease or allergy), radioisotope imaging was performed. The definition of “early” was during the same postoperative hospitalization period.

### Definition of the graft event

Graft events were classified into 2 categories. When intraoperative flow insufficiency required conversion to a SVG, this was defined as a clinical graft event. Radiological graft events were defined as either (1) graft occlusion confirmed by CT angiography, or (2) stenosis at any segment of the graft or poor peripheral contrast enhancement demonstrated by imaging, accompanied by evidence of myocardial ischemia on radioisotope imaging.

### Definition of major adverse cardiac and cerebrovascular events

Major adverse cardiovascular events (MACCE) was defined as cardiovascular death (including sudden death), non-fatal myocardial infarction, non-fatal stroke, hospitalization for heart failure, or repeat coronary revascularization.

### Statistical analysis

Statistical analyses were performed using R software (version 4.2.2; R Foundation for Statistical Computing, Vienna, Austria). Continuous variables are presented as median and interquartile range, calculated using the inclusive method. Analysis of the difference in RIMA diameter was performed using Mann-Whitney test. The difference of mean graft flow and PI between left and right internal mammary arteries (IMAs) were analysed using the Wilcoxon signed-rank test. The duration of follow-up was reported as a conventional median, without estimation using the reverse Kaplan-Meier method. The cumulative incidence of graft event was analysed using the cumulative incidence function (CIF) based on Aalen-Johansen estimator, with death considered as a competing risk. Pointwise 95% confidence intervals were calculated based on the variance of the point estimates, applying the delta method with a complementary log-log transformation. Long-term outcomes were analysed using the Kaplan-Meier method with 95% confidence intervals.

## RESULTS

### Baseline patient characteristics

Baseline patient characteristics are summarized in **Table 1**. The median age was 64 (54-68) years, with a male-to-female ratio of 135:10. The average number of distal anastomoses per patient was 3 (3-4). Coronary artery bypass grafting was performed on-pump in 136 cases and off-pump in 9 cases. The median follow-up duration was 48 (14-99) months. Detailed surgical data are presented in **Table 2**.

**Table 1.** Basement Patient Characteristics

Age (years)	64 (54-68)
Male	135 (93%)
Height (cm)	167 (163-172)
Weight (kg)	67 (60-75)
Diabetes mellitus	82 (56%)
Hypertension	99 (68%)
Dyslipidaemia	106 (73%)
COPD	13 (9.0%)
Chronic kidney disease	38 (26%)
Renal replacement therapy	11 (7.6%)
Peripheral arterial disease	19 (13%)
Preoperative mean LVEF (%)	58 (45-67)
Diagnosis	
Stable angina	64 (44%)
Acute coronary syndrome	56 (39%)
Old myocardial infarction	22 (15%)
Silent myocardial ischaemia	3 (2.1%)
Emergent cases	9 (6.2%)
Number of total distal anastomosis	3 (3-4)
Number of RIMA distal anastomosis	1 (1-1)
Number of on-pump cases	136 (94%)
Follow-up duration (months)	48 (14-99)

Abbreviations: COPD, chronic obstructive pulmonary disease; LVEF, left ventricular ejection fraction; RIMA, right internal mammary artery.

**Table 2.** Intra and Postoperative Data

RIMA distal anastomosis site	139 (96%)
Circumflex branch	
Left anterior descending artery	3 (2%)
Right coronary artery	3 (2%)
RIMA graft flow (mL/min)	42 (25-55)
RIMA pulsatility index (PI)	1.8 (1.4-2.3)
RIMA Diastolic filling (%)	65 (60-69)
LIMA-LAD graft flow (mL/min)	45 (33-66)
LIMA-LAD pulsatility index (PI)	1.8 (1.4-2.2)
LIMA-LAD diastolic filling (%)	71 (66-75)
Aortic cross-clamp time (min)	142 (120-170)
Operation time (min)	489 (437-553)
Postoperative peak creatine kinase (ng/mL)	29(21-36)
Revision cases	2 (1.4%)
Newly hemodialysis cases	1 (0.7%)
Cerebrovascular accident cases	1 (0.7%)
Surgical site infection cases	
Superficial	3 (2.1%)
Deep	6 (4.1%)

Abbreviations: LAD, left anterior descending artery; LIMA, left internal mammary artery; LVEF, left ventricular ejection fraction; MACCE, major adverse cardiac and cerebrovascular event; RIMA, right internal mammary artery.

### RIMA configuration

The RIMA was anastomosed to the circumflex branch in 139 cases (96%), to the RCA in 3 cases (2%), and to the LAD in 3 cases (2%). A single peripheral anastomosis was performed in 132 cases (91%), while 13 cases (9%) received 2 distal anastomoses—all of which targeted the circumflex branch.

### RIMA diameter

The median proximal diameter of the RIMA was 2.6 (2.3-2.8) mm in all cases. When stratified by sex, 2.6 (2.3-2.8) mm in males and 2.3 (2.0-2.6) mm in females, demonstrating a statistically significant difference (*P* = .002).

### Graft flow

The median flow and PI of the RIMA were 42 mL/min (25-55) and 1.8 (1.4-2.3), respectively. For the LIMA-LAD graft, median flow and PI were 45 mL/min (33-66) and 1.8 (1.4-2.2), respectively. Detailed flow data are presented in **Table 2**.

A significant difference was observed in flow between the 2 grafts (*P* = .001), whereas the difference in PI was not statistically significant (*P* = .932).

### Early outcomes

Two patients (1.4%) died during the early postoperative period due to fatal arrhythmia. One patient (0.7%) required initiation of dialysis, 2 (1.4%) underwent surgical revision for bleeding, 1 (0.7%) experienced a cerebrovascular accident, and surgical site infections (SSI) occurred in 9 cases (6.2%), 3 (2.1%) superficial and 6 (4.1%) involving the deep sternal wound.

### Long-term survival

**Figure 2.**
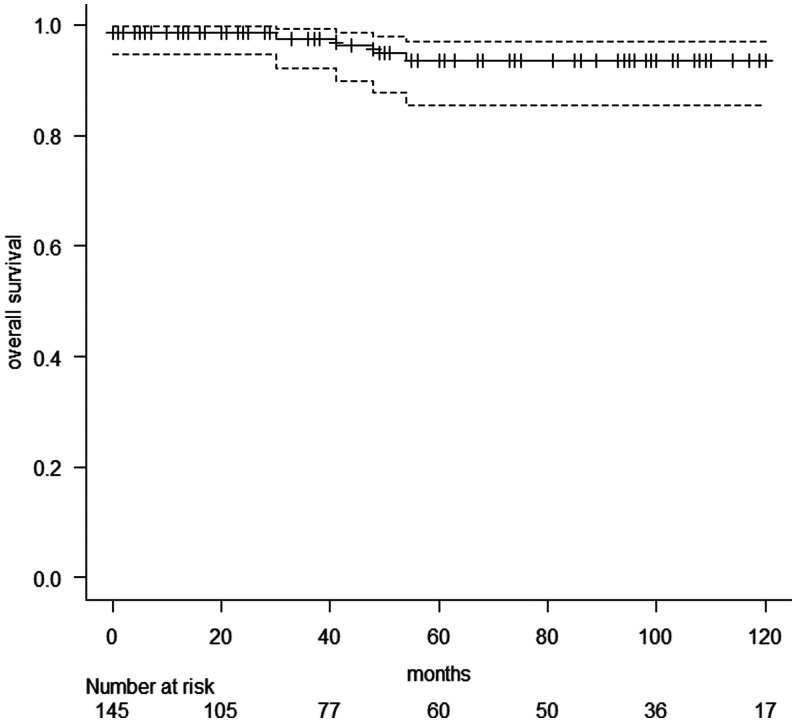
Overall Survival Rate

During the follow-up period, 6 deaths (4.1%) were recorded including the 2 (1.4%) early postoperative deaths. The other causes of death included malignancy (*n* = 2 [1.4%]), aspiration pneumonia (*n* = 1 [0.7%]), and cardiopulmonary arrest of unknown aetiology (*n* = 1 [0.7%]). The 10-year overall survival rate was 93.4% (95% confidence interval: 85.6%-97.1%, **Figure 2**).

### Imaging follow-up rate

The imaging follow-up rate was 93% at 1 year postoperatively, with 7 patients (4.7%) lost to follow-up and 3 patients (2%) not undergoing imaging among 143 surviving or presumed-surviving patients (excluding 2 confirmed deaths). At 5 years, the rate was 77%, with 15 patients (10%) lost to follow-up and 3 (2%) not undergoing imaging among 77 patients, excluding 62 who had not yet reached the 5-year point and 6 confirmed deaths. At 10 years, 105 patients (72%) had not yet reached the follow-up point. Among the remaining 34 patients (excluding 6 deaths), 11 were lost to follow-up and 2 did not undergo imaging, resulting in a 62% imaging follow-up rate.

### Clinical and radiological graft events

**Figure 3.**
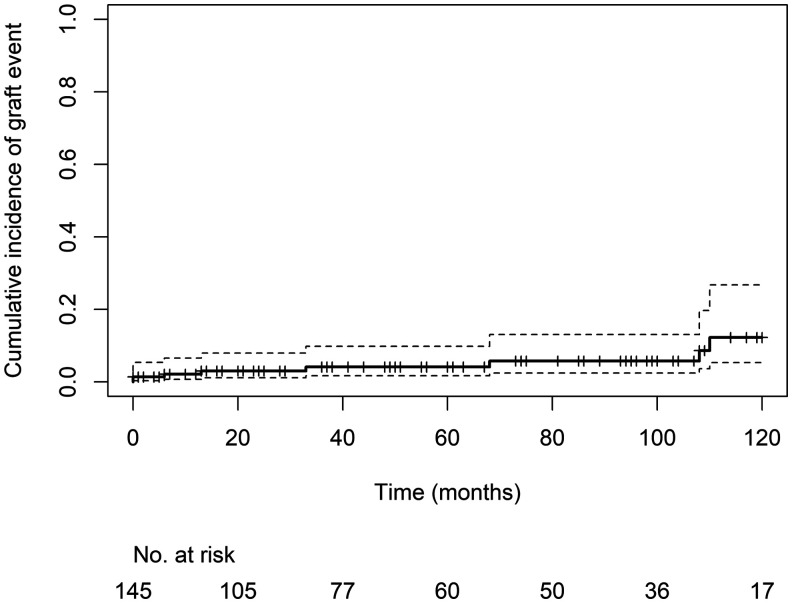
Cumulative Incidence of Graft Events

Graft-related events were observed in 7 patients (4.8%). One patient (0.7%) required conversion to SVG grafting due to poor flow in the free RIMA. Two cases (1.4%) of early graft occlusion were identified on postoperative CT, and both underwent percutaneous coronary intervention (PCI) targeting the circumflex branch. Additionally, PCI was performed in 2 patients (1.4%) due to proximal RIMA stenosis, including the anastomotic site, as detected on CT at 1 year postoperatively and during an additional examination at 3 years postoperatively. Two further events were observed in 1 patient at 5 years on CT and in another patient at 10 years on CT. At 10-year follow-up, cumulative incidence of graft events with death treated as a competing risk was 12.3% (95% confidence interval: 5.3%-26.8%, **Figure 3**).

### Major adverse cardiac and cerebrovascular events (MACCE) events

**Figure 4.**
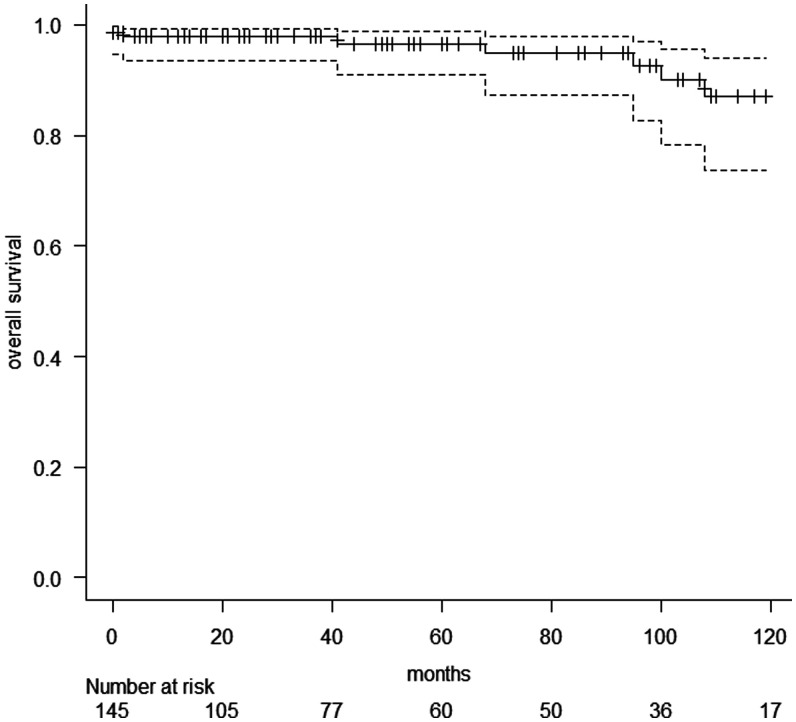
MACCE-Free Rate

Major adverse cardiovascular events occurred in 11 (7.6%) patients during the follow-up period. These included 2 early deaths (1.4%), 1 sudden death (0.7%), 2 PCI procedures for RIMA (1.4%), 5 additional coronary revascularizations (3.4%), and 1 stroke (0.7%). The 10-year MACCE-free rate was 82.0% (95% confidence interval: 63.9%-91.6%, **Figure 4**).

## DISCUSSION

The IMA has been recognized for its vasodilatory properties and anti-atherosclerotic effects, primarily mediated by endothelium-derived nitric oxide production.^14^ These characteristics make it a valuable conduit in CABG. Additionally, skeletonization of the IMA is hypothesized to induce sympathetic denervation, thereby reducing the risk of vasospasm.^15^

Optimal utilization of RIMA is considered essential for achieving favourable long-term outcomes following CABG. However, its application remains controversial. Aranda-Michel et al^16^ reported no significant difference in long-term outcomes between free and *in situ* RIMA grafts, while Magruder found no variation in mortality or revascularization rates based on RIMA configuration.^17^ These findings, however, may be influenced by patient selection bias and surgeons’ preference. Furthermore, from a haemodynamic perspective, shorter grafts are associated with reduced energy loss and improved flow volume.^18^

According to these considerations, we advocate for the use of LIMA to revascularize the LAD, as it remains the most reliable approach. We further propose that employing the RIMA as a free graft for revascularization of the remaining coronary territories offers greater flexibility in graft design while maintaining favourable long-term outcomes. Hayashi et al^19^ demonstrated superior outcomes when RIMA was used as a free graft, and Assi reported that free RIMA grafts yielded long-term results comparable to those of LIMA.^10^ These findings support the potential of free RIMA to enhance CABG outcomes when appropriately applied.

Given the smaller diameter of RIMA compared to venous grafts, conventional anastomotic techniques may increase the risk of stenosis. In our cohort, the median diameter of the proximal RIMA was 2.6 mm. Hefel et al,^20^ in a cadaveric study, reported that at the level of the fourth rib, the RIMA diameter slightly exceeded that of LIMA, measuring 2.00 mm in males and 1.88 mm in females. Karaman et al,^21^ using multidetector CT, found a median IMA diameter of 2.5 mm at its origin, with no significant sex-based differences. These findings are consistent with our measurements and confirm the relatively small caliber of the RIMA.

Loop et al^8^ reported a patency rate of 77% for free RIMA grafts within 18 months postoperatively, attributing early failures to limited technical experience with aortic anastomosis. Addressing the challenges of proximal RIMA anastomosis, several techniques have been proposed. Ito et al^22^ described a foldback method involving side-to-side anastomosis to the aorta, though this approach necessitates a longer suture line. Other methods involve composite grafting using venous or arterial conduits.^23^^,^^24^

Our technique offers distinct advantages. It eliminates the need for additional grafts and produces a dome-shaped bulge resembling a cobra head. The aortic anastomotic length, created using a standard 4 mm punch, approximates 12 mm (4 × π). A 10-mm cutback yields a suture line length of graft diameter  ×  π + 10 mm, providing a balanced anastomotic margin.

Many reports in the literature describe configurations in which the RIMA is anastomosed to the SVG after a proximal anastomosis to the ascending aorta, or employ other proximal modifications.^22–24^ These studies, which have comparable results to ours, have demonstrated superior outcomes compared with those performed without modification during the earlier era of the procedure.^8^ We suggest that not only Pouch technique, but also other surgical modifications of the proximal anastomosis have contributed to improved clinical applicability of free RIMA grafts.

Pouch technique presents certain tips and pitfalls. The fixed angle between the graft and aorta may complicate anastomosis to the RCA, particularly when the graft traverses the anterior surface of the right ventricle. In such cases, positioning the heel of the anastomosis towards the right atrium or selecting the aortic anastomotic site to more distal side may help prevent kinking. From a technical standpoint, the dome ceiling corresponds to the heel portion of the anastomosis. When employing the parachute technique, care must be taken to avoid excessive suture bites on the aortic side, which may lead to bleeding.^11^ In our series, 2 cases of stenosis were observed at the pouch site. We believe that meticulous attention to these technical nuances is essential for ensuring safe and durable anastomosis.

### Study limitations

The primary limitation of this study is the absence of a control group, which precluded us from determining whether Pouch technique directly contributed to the improved graft patency. Due to practical and ethical constraints, conducting a randomized controlled trial to address this issue is not feasible. Comparative observational approaches—such as cohort comparison, propensity score matching, or inverse probability weighting—could not be applied to this dataset. To contextualize our findings, we compared our outcomes with previously published results of free grafts without this modification, which represents the most appropriate external benchmark for a single-arm study.

In addition, this study was conducted at a single centre and employed a retrospective design. The 10-year follow-up rate for the imaging studies was limited by the small cohort of patients, which may have affected the robustness of the long-term outcome analysis. Although the inclusion and exclusion criteria, including a structurally healthy ascending aorta, are generally considered appropriate, the possibility of selection bias cannot be entirely ruled out.

The predominance of male patients and high proportion of bypass grafts to the circumflex branch in this case series may also limit the generalizability of the findings.

Our operative time was prolonged, indicating that technical improvements are needed, and that the extended duration may have increased the risk of perioperative myocardial infarction and potentially affected postoperative cardiac function.^25^ However, incidence of perioperative myocardial infarction could not be assessed because cardiac troponin levels, the key marker for PMI,^26^ were unavailable.

## CONCLUSION

The combination of free RIMA graft and Pouch technique, a dome-like proximal configuration, demonstrated favourable long-term outcomes with a low incidence of proximal anastomotic complications.

## Data Availability

Data are available in a repository and can be accessed via a DOI link.

## ABBREVIATIONS

CABG: Coronary artery bypass grafting
IMA: Internal mammary artery
LAD: Left anterior descending artery
LIMA: Left internal mammary artery
MACCE: Major adverse cardiac and cerebrovascular events
PI: Pulsatility index
RCA: Right coronary artery
RIMA: Right internal mammary artery
SVG: Saphenous vein graft
